# Dynamic changes of synergy relationship between lncRNA and immune checkpoint in cancer progression

**DOI:** 10.1093/bib/bbaf370

**Published:** 2025-07-29

**Authors:** Chenyu Liu, Qianyi Lu, Jian Li, Di Wang, Zhuoru Wang, Wenli Chen, Yakun Zhang, Caiyu Zhang, Yue Gao, Shangwei Ning

**Affiliations:** College of Bioinformatics Science and Technology, Harbin Medical University, No. 157, Baojian Road, Nangang District, Harbin City, Heilongjiang Province, China; College of Bioinformatics Science and Technology, Harbin Medical University, No. 157, Baojian Road, Nangang District, Harbin City, Heilongjiang Province, China; College of Bioinformatics Science and Technology, Harbin Medical University, No. 157, Baojian Road, Nangang District, Harbin City, Heilongjiang Province, China; College of Bioinformatics Science and Technology, Harbin Medical University, No. 157, Baojian Road, Nangang District, Harbin City, Heilongjiang Province, China; College of Bioinformatics Science and Technology, Harbin Medical University, No. 157, Baojian Road, Nangang District, Harbin City, Heilongjiang Province, China; College of Bioinformatics Science and Technology, Harbin Medical University, No. 157, Baojian Road, Nangang District, Harbin City, Heilongjiang Province, China; College of Bioinformatics Science and Technology, Harbin Medical University, No. 157, Baojian Road, Nangang District, Harbin City, Heilongjiang Province, China; College of Bioinformatics Science and Technology, Harbin Medical University, No. 157, Baojian Road, Nangang District, Harbin City, Heilongjiang Province, China; College of Bioinformatics Science and Technology, Harbin Medical University, No. 157, Baojian Road, Nangang District, Harbin City, Heilongjiang Province, China; College of Bioinformatics Science and Technology, Harbin Medical University, No. 157, Baojian Road, Nangang District, Harbin City, Heilongjiang Province, China

**Keywords:** immunotherapy, lncRNA, immune checkpoint, synergy relationship

## Abstract

In the battle between tumors and the immune system, immune evasion based on immune checkpoints (ICPs) is a critical mechanism for tumor progression. Long noncoding RNAs (lncRNAs) are key players in tumorigenesis and immune responses; however, the mechanisms underlying the synergistic relationship between lncRNAs and ICPs in cancer progression remain poorly understood. Manually curated ICPs and high-confidence lncRNA-messenger RNA (mRNA) interactions were integrated via a protein-protein interaction (PPI) network to construct an initial set of lncRNA-ICP pairs. Stage-specific synergy scores were then performed and used to identify stage-specific synergistic pairs for each cancer type. Our findings indicate that several key genes, including MALAT1 and CRNDE, are widely involved in cancer progression and exhibit various patterns in multiple cancers. Genes within the lncRNA-ICP synergy network were associated with the dynamic changes of immune cells during cancer progression, and these relationships remain relatively stable across different cancers and stages. The relationships of the synergistic pairs we identified demonstrate consistency with spatial transcriptomics data in skin cutaneous melanoma. Notably, the overall expression of genes identified in Stage 4 could significantly differentiate patients’ survival outcomes. Moreover, the genes we identified could distinguish patients’ responses to immunotherapy.

## Introduction

In 2022, nearly 20 million new cancer cases were reported globally, with ~9.7 million deaths attributed to cancer. About 1 in 5 people developed cancer during their lifetime, while ~1 in 9 men and 1 in 12 women succumbed to the disease. In 177 out of 183 countries, cancer ranked among the top three leading causes of death for individuals aged 30–69 [1]. Cancer remains a significant public health challenge, posing a serious threat to human health worldwide.

During cancer development and progression, tumors undergo evolution and may employ various mechanisms to evade immunosurveillance and suppress antitumor immune responses. One primary mechanism of tumor immune escape is the engagement of immune checkpoint (ICP) pathways [2]. In recent years, immunotherapy strategies that block ICP signaling between tumor cells and immune cells, thereby lifting immunosuppression and enhancing the body’s antitumor immune response, have made significant progress. Although multiple types of cancers have shown sustained clinical responses to immunotherapy, most patients receiving ICP inhibitors (ICIs) do not benefit from the treatment, and the underlying mechanisms are still not fully understood [3].

LncRNAs are a class of noncoding RNAs with a length of over 200 nucleotides, and they are widely involved in various biological processes. Recently, accumulating evidence has highlighted the critical role of lncRNAs in modulating immune system functions, with potential implications for the efficacy of immunotherapeutic interventions. Joseph *et al*. found that the expression level of NEAT1 is associated with the response of melanoma patients to ICP blockade, which may be linked to the upregulation of the interferon pathway [4]. Zhang *et al*. developed a machine learning model based on 16 immune cell–associated lncRNAs to identify LGG populations that may benefit from immunotherapy [5]. Shao *et al*. demonstrated that treatment with CD55/CD59-neutralizing antibodies or mutation of the LINC00973 promoter activates the complement system and CD8+ T cells, thereby inhibiting tumor growth. Moreover, the combination of anti-CD55/CD59 and anti-PD-1 antibody treatments resulted in a synergistic tumor-suppressive effect [6]. These studies suggest that lncRNAs may exert regulatory effects on ICPs and related pathways, potentially influencing the clinical outcomes of immunotherapy.

In this study, multiple resources were systematically collected and integrated to infer potential relationships between lncRNAs and ICPs. By employing stage-specific scoring, synergistic pairs that exhibit significant co-expression at specific stages of each cancer type were identified. As a consequence, the evolution of the synergistic relationship between lncRNAs and ICPs during cancer progression was characterized.

## Materials and methods

### Sources and scope of cancer data

Gene expression and clinical data for 15 cancer types were obtained from The Cancer Genome Atlas (TCGA, https://portal.gdc.cancer.gov). Transcriptome data of melanoma patients receiving immunotherapy were obtained from the Tumor Immunotherapy Gene Expression Resource (TIGER, http://tiger.canceromics.org). Single-cell transcriptome data of melanoma brain metastasis samples and lung adenocarcinoma samples were accessed via Curated Cancer Cell Atlas (3CA, https//www.weizmann.ac.il/sites/3CA/); spatial transcriptome data of melanoma brain metastasis patients [7] were accessed via Gene Expression Omnibus (GEO, https://www.ncbi.nlm.nih.gov/geo/).

### Construction of initial long noncoding RNA–immune checkpoint interaction network

Clinical and experimentally verified ICP genes were manually collected from literature included in PubMed, handbooks, or instructions of websites from multiple companies [8]. To identify lncRNA-mRNA interaction networks, starBase [9], RAID v2.0 [10], and NPInter [11] databases were used. Finally, 81 186 nonredundant and validated lncRNA-mRNA pairs were identified [12]. Protein–protein interaction (PPI) relationships were obtained from the STRING database [13]. Only samples from stages with >10 cases for each cancer type were included in the analysis. Firstly, proteins that interact with ICPs were obtained from the PPI network, and ICPs and interacting proteins are mapped to the lncRNA-mRNA pairs. Each pair was sequentially evaluated, and only those in which at least one of either the lncRNA or the mRNA exhibits differential expression across stages, as determined by the Kruskal–Wallis test, were retained for further analysis. Then, the lncRNA-mRNA interaction network centered around ICPs was established.

### Identification of stage-specific synergy long noncoding RNA–immune checkpoint pairs

The Pearson correlation for each pair in the initial lncRNA-ICP interaction network at different cancer stages was calculated; then, the stage-specificity lncRNA-ICP synergy score (ssLIss) of each pair in each cancer was defined:


\begin{align*} ssLIss=&\frac{\sum \left(1-| Adjusted\ {PCC}_{s_j}|/\max \left(| Adjusted\ PCC|\right)\right)}{N-1}\\&\ast \frac{\max \left(| Adjusted\ PCC|\right)}{0.75} \end{align*}

where $Adjusted\ {PCC}_{s_j}$represents the adjusted Pearson correlation coefficient of lncRNA-ICP pair in stage $j$,$Adjusted\ PCC$represents adjusted Pearson correlation coefficients of all stages, $N$ represents the number of stages in one cancer.


\begin{align*} Adjusted\ {PCC}_{s_j}=\left\{\begin{array}{@{}l} correlation\ coefficient,\ p<0.05\\{} 0\kern8.75em ,\ p\ge 0.05\end{array}\right. \end{align*}

The significance of ssLIss was assessed by performing 1000 random perturbation trials, using a threshold of 0.05. Pairs with significantly high ssLIss were defined as stage-specific synergy lncRNA-ICP pairs, and the stage with the highest |Adjusted PCC| was chosen as the specific stage.

### Enrichment analysis of functional long noncoding RNAs for long noncoding RNAs in synergy network

The cumulative hypergeometric test was used to determine whether the lncRNAs we identified significantly overlap with the functional lncRNAs recorded in the database; the *P*-value for the enrichment was calculated as follows:


\begin{align*} \mathrm{P}=1-\sum_{i=0}^{k-1}\frac{\left(\genfrac{}{}{0pt}{}{m}{i}\right)\left(\genfrac{}{}{0pt}{}{N-m}{n-i}\right)}{\left(\genfrac{}{}{0pt}{}{N}{n}\right)} \end{align*}

where $N$ represents the total number of lncRNAs in TCGA, $m$ represents the number of functional lncRNAs in the database, $n$ represents the number of specific stages lncRNAs in synergy networks across cancers, and $k$ represents the number of intersections of $m$,$n$. In the PubMed search, conducted using the R package “RISmed” [14], 𝑚 represents the number of lncRNAs that co-occurred with the keyword “early stage” or “metastasis” in at least one publication.

### Enrichment analysis of Reactome pathways

Reactome pathway enrichment analysis was performed using the R package “clusterProfiler” [15]. Significantly enriched pathways with FDR < 0.05 were filtered, and the corresponding topics of pathways were collected from the Reactome website [16].

### Identification of cell type–related synergistic genes

The enrichment scores of multiple cell types for each sample were obtained using the xCell algorithm through the “IOBR” R package with default parameters. Specifically, the expression profile we provided to the xCell method was the quality-controlled version of the whole-transcriptome gene expression data. Subsequently, Spearman correlations between the enrichment scores of each cell type and synergistic genes were calculated separately at different stages of each cancer type. Genes associated with cell types at specific stage were identified using a threshold of *P* < .05.

### Single-cell transcriptome preprocessing and cell type annotation

scRNA-seq data from 14 lung adenocarcinoma samples (*n* = 32 493 cells) [17] and 32 skin cutaneous melanoma (SKCM) samples (*n* = 136 973 cells) [18] were obtained from the 3CA database. For the SKCM data, the scaled data and cell type annotations provided by the 3CA database were utilized, with only brain metastasis samples retained for analysis. For the lung adenocarcinoma data, re-normalization and re-annotation were performed to achieve a finer resolution of cell type classification, as required for the objectives of this study. After quality control filtering—excluding cells with >10% mitochondrial gene expression, fewer than 500 detected genes, fewer than 1000 UMI counts, and suspected doublets, identified by co-expression of markers from multiple lineages within the same cluster. Finally, a total of 31 379 high-quality cells were retained. Data processing followed the Seurat pipeline, including normalization, variable feature selection, scaling, and principal component analysis, with the top 30 principal components selected. Batch effects across samples were corrected using the Harmony algorithm. Uniform manifold approximation and projection (UMAP) was applied for dimensionality reduction, and cell clustering was performed using the FindNeighbors and FindClusters functions, with a resolution set to 0.8. Cell type annotation was based on canonical marker gene expression. Following the identification of major cell types, a more detailed annotation of immune cell subsets was performed. Specifically, T cells, natural killer (NK) cells, B cells, and macrophages were further classified into subpopulations.

### Identifying significant co-occurrence and mutually exclusive relationships in spatial transcriptomics samples

The Seurat R package [19] was first used to preprocess and normalize single-cell and spatial transcriptomics data. Then, based on the single-cell data, enrichment scores for different cell types in the spatial transcriptomics spots were estimated. Co-occurrence and mutual exclusivity between lncRNA-mRNA pairs or gene–cell type associations were determined using Fisher’s exact test:


\begin{align*} \mathbf{P}=\frac{\left(\genfrac{}{}{0pt}{}{\boldsymbol{a}+\boldsymbol{b}}{\boldsymbol{a}}\right)\ast \left(\genfrac{}{}{0pt}{}{\boldsymbol{c}+\boldsymbol{d}}{\boldsymbol{c}}\right)}{\left(\genfrac{}{}{0pt}{}{\boldsymbol{n}}{\boldsymbol{a}+\boldsymbol{c}}\right)} \end{align*}

In this context, *a* represents the number of spots where both two genes or one gene and a specific cell type are expressed together, while *b* and *c* represent the number of spots where one is expressed and the other is not, *d* denotes the number of spots where neither is expressed, and *n* is the total number of spots in a spatial transcriptomics sample. Using a significance threshold of 0.05, co-occurrence or mutual exclusivity between the two entities was determined based on the ratio, which is defined as (*a* + *d*)/(*b* + *c*).

### Survival analysis

Clinical information of patients was obtained from TCGA database. For patients labeled as “Dead,” the variable “days_to_death” was used, while for patients labeled as “Alive,” “days_to_last_follow_up” was selected. Genes identified in Stage 4 for each cancer type were selected and further filtered to retain those that were either up-regulated or down-regulated relative to samples from other stages. For each cancer type, the pathway level analysis of gene expression (PLAGE) method was then employed to score the cancer samples based on the identified up-regulated and down-regulated gene sets. The samples were divided into high and low score groups according to the median score, and survival differences between the groups were subsequently compared.

### Construction and validation of classifier models for distinguishing immunotherapy response

Two melanoma immunotherapy data from the TIGER database were used to build and validate the model for demonstrating the ability of synergistic genes to predict immunotherapy response. The Melanoma-PRJEB23709 cohort [20], which includes 40 responders and 33 nonresponders, with 80% of the samples used for model training and 20% for model testing. We validated the model using GSE115821 as an independent dataset, which includes 2 responders and 10 nonresponders. For this analysis, only pretreatment samples were included. Classifier models were constructed and refined using the R package “mlr3” [21]. All synergistic genes identified in SKCM were included as candidates, and feature selection was performed using the genetic search algorithm. To ensure a relative balance between responder and nonresponder samples, stratified sampling was applied in both hold-out validation and 5-fold cross-validation splits. For each classifier, 100 iterations of feature selection followed by 5-fold cross-validation were conducted. Only classifier–feature combinations achieving an average AUC exceeding 0.75 across the 5 folds were retained for further validation. Based on AUC performance in both the 5-fold cross-validation and the predivided validation set, the optimal model was identified and applied to the independent validation set for evaluation.

### Statistical analysis

Network clustering was performed using the Glay algorithm from the clusterMaker plugin in Cytoscape software. Kaplan–Meier survival analysis was conducted to evaluate overall survival differences between the two groups, which are defined by synergy signature score, utilizing the R package “survival.” Single-sample enrichment scores for synergy genes in cancer samples were calculated using the PLAGE method implemented in the R package “GSVA.”

## Results

### Construction of stage-specific long noncoding RNA–immune checkpoint synergistic network in cancers

**Figure 1:**
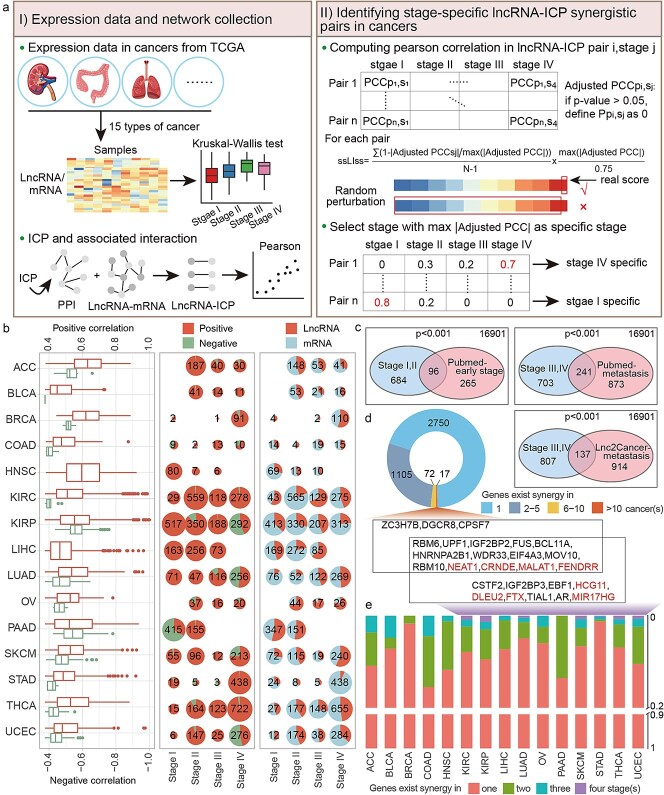
Identification and overview of stage-specific synergistic pairs in multiple cancer types. (a) Workflow for identifying stage-specific synergistic pairs in cancers. (b) Distribution of correlation coefficients (left), numbers of positive and negative correlations identified at each stage (middle), and numbers of lncRNAs and genes involved in synergistic pairs at each stage (right) in cancers. (c) Venn plot showing the overlap between lncRNAs involved in synergistic pairs and functional genes identified through database searches. (d, e) Distribution of synergistic genes across various cancer types (d) and stages (e), with lncRNAs highlighted in red.

To explore the synergistic roles of lncRNAs and ICPs in cancer progression, lncRNA-ICP correlation coefficients were calculated at different stages based on RNA-seq profiles of 15 cancers from TCGA (namely, ACC, BLCA, BRCA, COAD, HNSC, KIRC, KIRP, LIHC, LUAD, OV, PAAD, SKCM, STAD, THCA, and UCEC). For each cancer type, stage-specific synergistic scores were calculated for each lncRNA-ICP pair to identify those exhibiting significantly enhanced co-expression at a particular stage (Fig. 1a, Materials and Methods). Compared to other cancer types, BLCA, COAD, LIHC, and STAD exhibited lower overall correlation strengths among synergistic pairs. In most cancers, the number of positively correlated pairs far exceeded that of negatively correlated pairs, and fewer lncRNAs were involved compared to protein-coding genes (Fig. 1b).

The lncRNAs identified at Stages I and II significantly overlapped with early-stage cancer-associated lncRNAs from PubMed text mining, whereas those identified at Stages III and IV significantly overlapped with metastasis-associated lncRNAs from both PubMed and the Lnc2Cancer database (Fig. 1c). The majority of lncRNAs and mRNAs exhibited both cancer-specific and stage-specific synergistic interactions, whereas a few, such as RBM6, IGF2BP2, MALAT1, and FENDRR, demonstrated synergy in >10 cancer types and in all four stages (Fig. 1d and e), suggesting their broad regulatory roles in multiple cancer progression.

Stage-specific synergistic networks were constructed based on these pairs. The degree distribution of nodes revealed that most nodes had low degrees, while a small subset exhibited high connectivity. Regardless of stage, MALAT1, NEAT1, CRNDE, and FENDRR consistently exhibited the highest degrees, indicating their widespread and stable regulatory roles in cancer progression (Supplementary Fig. 1).

### Long noncoding RNA–immune checkpoint synergy modules reflect cancer heterogeneity

**Figure 2:**
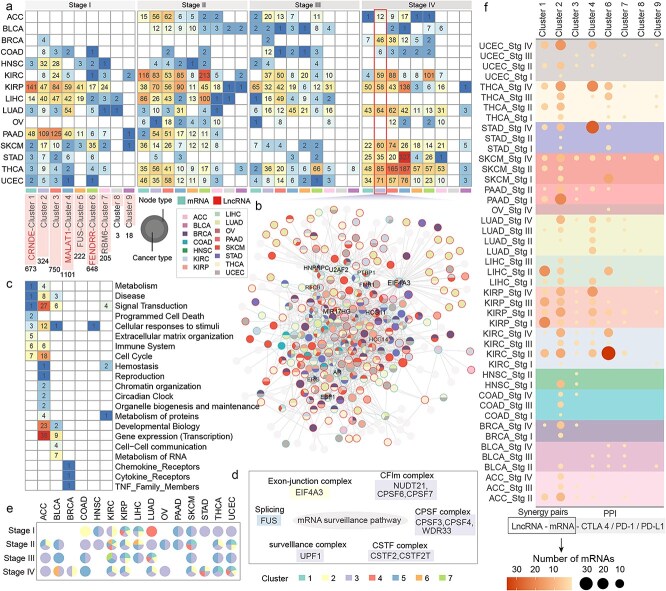
Distribution and functional insights of synergistic modules across cancer types. (a) Distribution of synergistic modules in different cancer types. (b) Mapping of Module 2 in Stage 4 cancers. (c) Functional enrichment results for Modules 1–7. (d) Mapping of modules to distinct functional regions within the mRNA surveillance pathway. (e) Modules mapped by synergistic genes identified across different stages and cancer types. (f) Distribution of genes interacting with PD-1, PD-L1, and CTLA-4 in cancer stages and modules.

The synergistic network was clustered into nine modules, including five lncRNA-centric modules and four mRNA-centric modules (Fig. 2a, Supplementary Figs 2–5). Different cancers at different stages exhibited varying preferences for these modules. For example, in Stage II, KIRC was broadly regulated by MALAT1, CRNDE, FENDRR, and NEAT1, while STAD at Stage IV was predominantly regulated by MALAT1. Wang *et al*. demonstrate that MALAT1 mediates glycolytic regulation to promote the progression of gastric cancer [22]. Compared to other modules, genes in Module 2 tend to participate in multiple cancer types (Fig. 2b), indicating genes in Module 2 may represent shared features of cancer development. Reactome pathway enrichment analysis revealed that genes in Module 2 were involved in diverse functions, including numerous signal transduction, gene expression regulation, and developmental biology pathways (Fig. 2c). Module 1 was specifically enriched in extracellular matrix–related functions, while Module 3 was associated with cell–cell communication and RNA metabolism.

The mRNA surveillance pathway was broadly involved across modules, with different modules occupying distinct functional units within the pathway. For example, EIF4A3 from Module 2 was involved in the exon-junction complex; FUS from Module 5 participated in splicing; and Module 3 contributed extensively to the CFIm complex, CPSF complex, CSTF complex, and surveillance complex (Fig. 2d). Compared to other cancer types, KIRC and KIRP exhibited changes across multiple modules and functional units of the pathway during cancer progression (Fig. 2e).

The number of synergistic genes associated with classical ICPs (PD-1, PD-L1, CTLA-4) across different cancers and modules was also compared (Fig. 2f). In UCEC, THCA, and STAD, the number of synergistic genes associated with classical ICPs increased progressively during cancer progression, while in KIRC and PAAD, more synergistic genes were observed at earlier stages. Notably, module 2 contained the broadest range of synergistic genes interacting with classical ICPs.

### Consistency and particularity of regulation patterns for immune checkpoint–long noncoding RNA synergistic relationships in cancers

**Figure 3:**
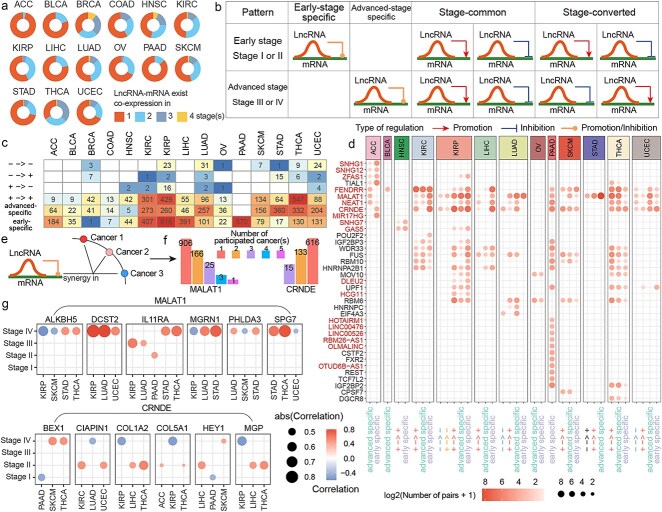
Expression patterns of synergistic pairs during cancer progression. (a) Number of stages in which synergistic pairs show significant correlations. (b) Four co-expression patterns of synergistic pairs based on correlation trends across stages. (c) Distribution of synergistic pairs with different co-expression patterns across cancers. (d) Genes primarily involved in synergistic pairs with distinct co-expression patterns. (e) Variable synergistic relationships of the same synergistic pair across cancer types. (f) Frequency of MALAT1 and CRNDE involvement in synergistic pairs across cancer types. (g) Synergistic relationships involving MALAT1 and CRNDE.

To better understand the characteristics of synergistic pairs across different stages of cancer progression, changes in the lncRNA-mediated regulation of mRNAs were investigated. Most synergistic pairs in pan-cancer exhibited co-expression relationships at only one stage, but a substantial proportion of pairs demonstrated co-expression at two or more stages (Fig. 3a). Based on their expression patterns across stages, synergistic pairs were classified into four categories: early-stage specific, advanced-stage specific, stage-common, and stage-converted (Fig. 3b). Stage-converted pairs were relatively rare in cancers, with most pairs falling into the other three categories. Early-stage specific synergistic pairs were more prevalent in ACC and LIHC, while advanced-stage specific pairs were more common in STAD. In THCA, stage common pairs dominated, whereas in KIRC, KIRP, LUAD, and UCEC, synergistic pairs were relatively evenly distributed across the three major patterns (Fig. 3c).

The key genes involved in each pattern within different cancers were further analyzed (Fig. 3d). In ACC, SNHG1, SNHG12, CRNDE, and MIR17HG were predominantly associated with early-stage specific pairs, while FENDRR, MALAT1, and NEAT1 were primarily involved in advanced-stage specific pairs. In other cancers, genes like FENDRR, MALAT1, NEAT1, CRNDE, FUS, WDR33, RBM10, HNRNPA2B1, and UPF1 were widely involved in multiple patterns of synergy. In contrast, genes such as IGF2BP2, TCF7L2, and OTUD6B-AS1 were primarily associated with specific patterns in PAAD.

Many synergistic pairs were found to exhibit co-expression in two or three stages, reflecting stage-specific associations (Supplementary Fig. 6). For instance, in KIRP, a significant proportion of synergistic pairs exhibited co-expression in both stage I and stage III. In THCA, most pairs demonstrated co-expression across stages I, III, and IV, while in KIRC, co-expression tended to occur in stages I and II. Conversely, some cancers exhibited strong stage specificity. For example, in ACC, almost no synergistic pairs showed co-expression in multiple stages.

A single synergistic pair may exhibit different correlation patterns across cancers (Fig. 3e). For instance, MALAT1 was involved in 195 synergistic pairs shared across two or more cancers, while CRNDE was involved in 148 such pairs (Fig. 3f). The synergy between MALAT1 and IL11RA was primarily observed in stage IV of STAD and THCA but occurred at earlier stages in KIRP, LUAD, and PAAD (Fig. 3g). The synergy between MALAT1 and ALKBH5 was consistently observed in stage four across four cancers but exhibited negative correlations in KIRP and SKCM, while showing positive correlations in STAD and THCA. Similarly, the correlations between CRNDE and COL1A2 or MGP were positive in stage II of LIHC and THCA but switched to negative in stage IV.

### Dynamic changes of long noncoding RNA–immune checkpoint synergistic network were shown for immune microenvironment in cancers

**Figure 4:**
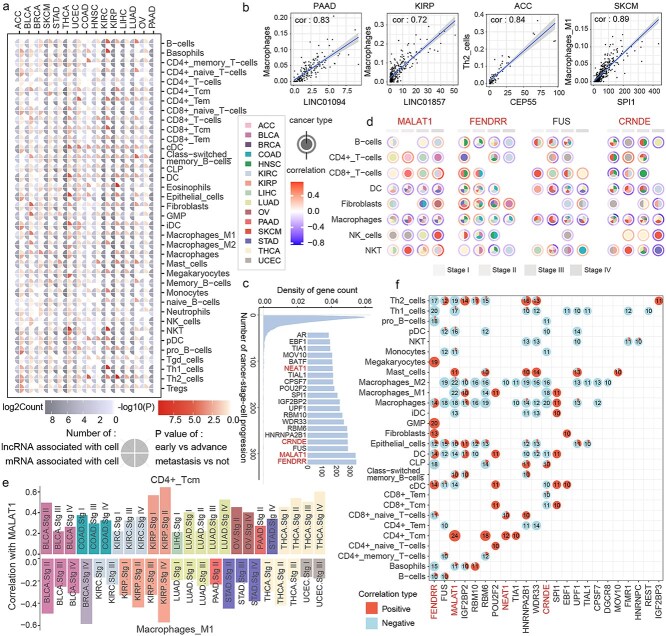
Relationship between genes in the synergistic network and the immune microenvironment. (a) *P*-values for differences in 37 cell type enrichment scores between early and late stages, as well as between metastatic and nonmetastatic stages, along with the numbers of lncRNAs and mRNAs associated with each score. (b) Selected genes are significantly associated with cell enrichment scores. (c) Number of genes involved in cancer-stage-cell processes. (d) Synergistic relationships between MALAT1, FENDRR, FUS, CRNDE, and specific cell types in different cancers. (e) Positive correlations between MALAT1 and CD4+ Tcm cells across cancer stages and negative correlations with M1 macrophages across all cancer types. (f) Numbers of positive and negative correlations between each gene and cell type across cancer stages.

To investigate the relationship between genes in the synergistic network and the tumor immune microenvironment, cell enrichment scores for common cell types of 15 cancer types were estimated using the xCell algorithm. Differences between early and advanced stages or metastatic and non-metastatic samples were assessed for each cell type, along with the number of lncRNAs and mRNAs associated with these cell types (Fig. 4a). Significant differences in the enrichment scores of various cell types were observed during cancer progression, such as NKT cells in THCA and KIRC. Significant correlations were also identified between genes in the synergistic network and cell enrichment scores (Fig. 4b).

The relationships between genes and cell enrichment scores at different stages and in cancer types were further examined. If a gene was correlated with the enrichment score of a specific cell type at a given stage in a particular cancer type, it was considered to participate in that cancer-stage-cell process. Genes such as FENDRR, MALAT1, FUS, and CRNDE were found to be involved in numerous cancer-stage-cell processes (Fig. 4c). A detailed investigation of their involvement in processes related to B cells and CD4+ T cells revealed that dendritic cells and macrophages were consistently correlated with MALAT1, FENDRR, FUS, and CRNDE across multiple cancers (Fig. 4d). Notably, a negative correlation of MALAT1 with M1 macrophages was observed across all cancers in which it was involved, while a positive correlation with CD4+ Tcm cells was observed (Fig. 4e). To further investigate the relationship between MALAT1 and CD4+ Tcm cells and M1 macrophages, the expression of MALAT1 in these two cell types across two stages was analyzed using the lung adenocarcinoma single-cell dataset (Supplementary Figs 7 and 8). The results showed that MALAT1 was significantly more highly expressed in CD4+ Tcm cells than in M1 macrophages.

To determine whether this condition was unique to these genes or represented commonly, the directionality of gene-cell correlations for all relationships observed in 10 or more cancer-stage combinations was analyzed (Fig. 4f). Highly consistent correlation patterns for gene-cell relationships were found in Th1 cells, pDCs, NKT cells, M2 macrophages, and CD4+ Tcm cells. To further explore the relationship between the genes and the positively and negatively correlated cell types, the expression of the genes in positively and negatively correlated cells was examined using the lung adenocarcinoma single-cell dataset. The results indicated that genes such as MALAT1, GSTO1, and NEAT1 had higher expression levels in the positively correlated cell types compared to those negatively correlated, while genes like THBS3, RASGRP2, and ABCA8 were rarely expressed in negative cells (Supplementary Fig. 9). This suggests the existence of stable gene–cell interactions that persist across different cancers and stages.

To investigate how cell-associated genes influence cellular functions, functional annotation of the genes associated with each cell type in each cancer type was performed using 17 immune-related pathways from the ImmPort [23] database. The results indicated that many of these genes are likely to influence immune cell functions by participating in cytokine and cytokine receptor pathways (Supplementary Fig. 10). Additionally, genes associated with dendritic cells in seven cancer types were mapped to the interleukin receptor pathway. We identified that there exist strong positive correlations between IL-7R and multiple immune cell types, such as CD4+ memory T cells and CD4+ naive T cells in SKCM and THCA. Studies demonstrate that IL-7/IL-7R deficiency severely impairs immune cell development, while increased IL-7 production promotes naive and memory T-cell survival [24].

### Synergistic immune checkpoint–long noncoding RNA pairs demonstrate consistency across the transcriptome and spatial transcriptome

**Figure 5:**
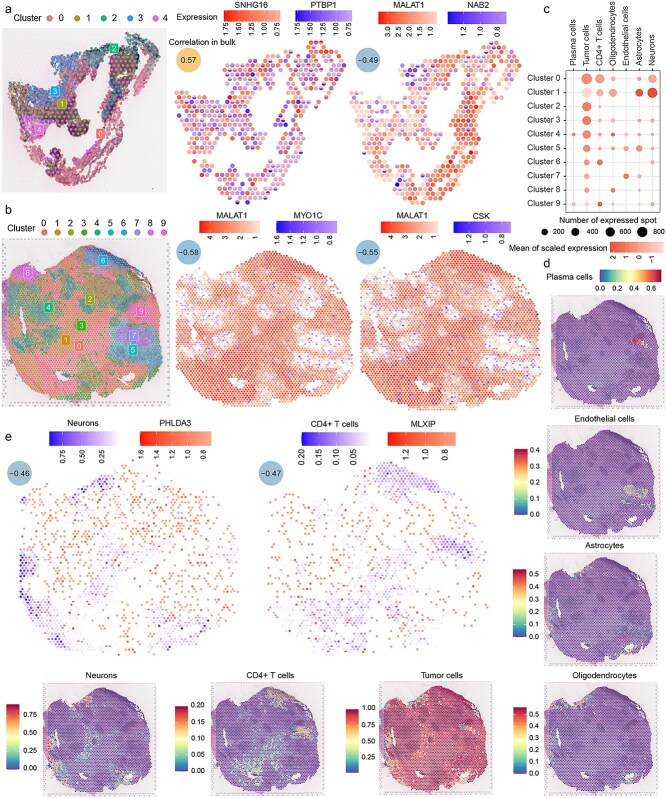
Spatial transcriptomics reveals lncRNA-mRNA synergistic relationships. (a, b) Two synergistic pairs show notable co-occurrence or mutual exclusivity tendencies in samples. (c) Bubble plot showing the expression and distribution of cell types across spatial clusters. (d) Spatial distribution of cell types across samples. (e) Genes and cell types that show significant negative correlations in bulk analysis exhibit distinct mutual exclusivity in their spatial expression and distribution.

The consistency of the identified synergistic relationships at the transcriptomic and spatial transcriptomic levels was validated by characterizing the spatial distribution of these relationships using a dataset of spatial transcriptomics from melanoma brain metastases. First, the spatial spots for each sample were clustered, with the first sample being divided into five clusters. In SKCM, the synergistic pair SNHG16 and PTBP1, identified in Stage 4 with a correlation coefficient of 0.57, was observed to co-express in a high proportion within the same spatial region in the pathological sections (Supplementary Fig. 11). In contrast, MALAT1 and NAB2, with a correlation coefficient of −0.49 in Stage 4, exhibited a significantly reduced co-expression within the same spatial region. MALAT1 was primarily expressed in Clusters 0, 2, and 4, while NAB2 was mostly expressed in Clusters 1 and 3, showing a mutually exclusive pattern (Fig. 5a).

The second sample contained 10 clusters. MALAT1 was widely expressed in Clusters 0, 1, 2, 3, 6, and 9, while MYO1C and CSK were predominantly expressed in Clusters 4, 5, and 7, as well as at the boundary regions between these clusters (Fig. 5b). The spots in Sample 2 were annotated using a single-cell melanoma brain metastasis dataset (Figs. 5c and d). Plasma cells were mainly enriched in Cluster 4, cancer cells were broadly distributed across the sections, CD4+ T cells were predominantly enriched in Clusters 0, 6, and 9, oligodendrocytes and astrocytes were primarily enriched at the peripheral regions, endothelial cells were concentrated in Clusters 5 and 7, and neurons were mostly enriched in Cluster 1.

In the Stage 4 samples of SKCM, PHLDA3 was negatively correlated with neurons, and MLXIP showed a negative correlation with CD4+ T cells. As observed in bulk, PHLDA3 and neurons, as well as MLXIP and CD4+ T cells, also demonstrated a clear tendency for mutual exclusion in spatial spots (Fig. 5e).

### Synergistic long noncoding RNA–immune checkpoint networks were involved in cancer prognosis and response for immunotherapy

**Figure 6:**
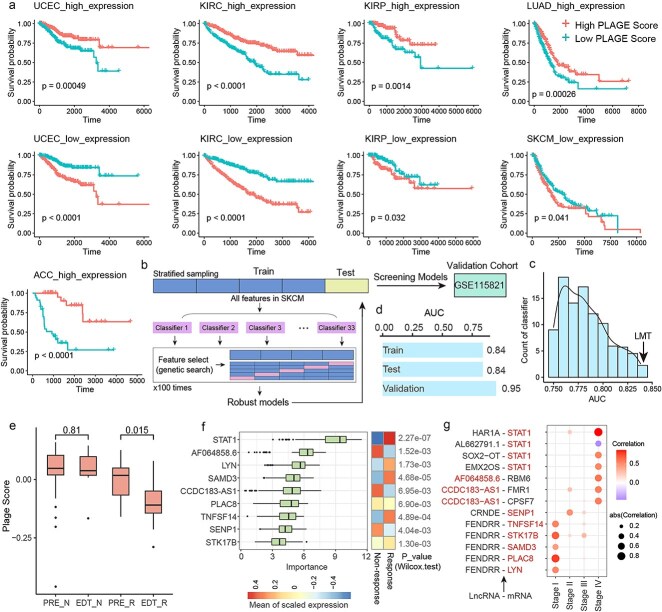
Genes in the synergistic network serve as prognostic markers. (a) PLAGE scores of synergistic genes in Stage 4 cancers differentiate survival outcomes. (b) Workflow for classifier construction and validation using the mlr3 package. (c) Effectiveness of the LMT model in distinguishing immune response. (d) Performance of the LMT model in training, validation, and independent test datasets. (e) Comparison of synergistic gene scores between responders and nonresponders pre- and post-treatment in SKCM. (f) Importance of driver genes identified by the Boruta algorithm in predicting immune response (left) and their expression in responder and nonresponder groups (right). (g) Synergistic pairs involving driver genes.

Differential analysis was performed on genes identified in Stage 4 in each cancer to select genes that exhibited significant expression differences compared to other stages. These differential genes were found to be associated with survival outcomes and could serve as potential biomarkers for prognostic stratification in multiple cancers. Compared to other cancer stages, genes highly expressed in Stage 4 were linked to poorer prognosis, with samples exhibiting higher enrichment scores for these genes showing significantly worse survival than those with lower enrichment scores. Similarly, genes with low expression in Stage 4 were associated with better prognosis, and samples with higher enrichment scores for these genes showed significantly better survival than those with lower scores (Fig. 6a).

To explore whether genes in the identified synergistic networks could distinguish response outcomes in cancer patients undergoing immunotherapy, a melanoma immunotherapy dataset was used for training and validating models, with another melanoma immunotherapy dataset, GSE115821, serving as an independent validation set. The mlr3 R package was utilized to construct and screen classifier models (Fig. 6b, Materials and Methods), ultimately identifying a robust and high-performing model, logistic model trees (LMT) (Fig. 6c). The model achieved an AUC of 0.84 in the training and validation sets and an AUC of 0.95 in the independent validation set (Fig. 6d). This model included four features—STAT1, AC139887.1, CCDC183-AS1, and AC022217.3, all of which were Stage 4–specific synergistic genes.

Changes in gene enrichment scores of the melanoma synergistic network before and after immunotherapy were compared between responder and nonresponder groups (Fig. 6e). In the nonresponder group, there was no significant difference in enrichment scores before and after immunotherapy. In contrast, the responder group exhibited a significant reduction in enrichment scores after immunotherapy, with enrichment scores in responders being consistently lower than those in nonresponders.

To identify the genes driving the response to immunotherapy, the Boruta method was used to detect significant driver features within the synergistic network, and a total of nine driver features were identified (Fig. 6f). The results revealed that STAT1 exhibited the most importance, followed by AF064858.6 and LYN. Significant differences in the expression of these features between responder and nonresponder groups in melanoma patients who received immunotherapy were observed. STAT1 is a key effector molecule of IFN-γ signaling, promoting immune cell recognition of tumors [25]. Skeate *et al*. reported that TNFSF14 could remodel the tumor microenvironment and synergize with ICIs to enhance the efficacy of cancer immunotherapy [26]. Scheuplein *et al*. demonstrated that inhibition of STK17B promotes the priming of naïve T cells and enhances the antitumor activity of anti-PD-L1 antibodies [27]. Fu *et al*. found that high SAMD3 expression in T cells is associated with better prognosis and increased T-cell infiltration [28]. Notably, STAT1 was involved exclusively in Stage 4–specific synergistic relationships within the melanoma network, whereas TNFSF14, STK17B, SAMD3, PLAC8, and LYN were all Stage 1–specific synergistic genes that synergized with FENDRR (Fig. 6g).

## Discussion

In recent years, cancer immunotherapy based on ICIs has achieved remarkable breakthroughs. ICP proteins such as PD-1 and CTLA-4, initiate signaling pathways that suppress T-cell function, thereby promoting T-cell exhaustion [29]. ICIs target these proteins to counteract tumor-mediated T-cell inhibition. Currently, drugs targeting CTLA-4 (ipilimumab, tremelimumab), PD-1 (pembrolizumab, nivolumab, cemiplimab), and its ligand PD-L1 (atezolizumab, avelumab, and durvalumab) have been introduced into clinical practice and have demonstrated considerable progress. However, the release of T-cell suppression induced by ICIs could result in a range of immune-related adverse events (irAEs), including but not limited to dermatologic toxicities, gastrointestinal toxicities, hepatitis, and endocrine disorders. Multiple mechanisms have been proposed to explain the occurrence of irAEs, but the precise pathophysiology remains incompletely understood [30]. Therefore, further investigation into ICP-related mechanisms and the development of strategies to mitigate treatment-associated adverse effects are critically needed.

Tumor progression is a dynamic and evolving process. The relative contributions of nutritional, metabolic, immune, and therapeutic factors to tumor evolution exhibit both spatial and temporal variations [31]. Recent studies have increasingly recognized lncRNAs as modulators of immunotherapy efficacy [5, 32, 33]. However, the systematic analysis of the collaborative relationship between lncRNAs and ICPs during cancer progression remains lacking. Here, we developed an integrated computational pipeline to characterize the dynamic synergistic relationships between lncRNAs and ICPs during tumor progression in multiple cancer types. Our findings reveal a complex relationship between lncRNAs and ICPs, highlighting the potential of these lncRNAs as biomarkers for predicting ICI responses in cancer patients.

In our analysis, the synergistic relationships mediated by lncRNAs in different cancer types and stages exhibit highly intricate. From a broader perspective, the synergistic interactions progressively increase from Stage 1 to Stage 2 and then to Stage 4, while Stage 3 seems to serve as a turning point, showing fewer interactions compared to both Stages 2 and 4. At the individual cluster level, KIRC is predominantly regulated by FENDRR in Stages 2 and 4. In Stage 2, it is additionally subjected to significant regulation by CRNDE; however, this regulatory influence by CRNDE is almost absent in Stage 4. For STAD, substantial regulation by MALAT1 is observed exclusively in Stage 4, whereas LUAD is consistently regulated by MALAT1 across all stages. These intricate regulatory dynamics may reflect the continuous evolution of the immune microenvironment during tumor progression. The relationship between synergistic genes and immune cells was investigated, along with the potential mechanisms by which these genes might influence immune cell function. However, the regulatory relationships between immune cells and molecules are highly complex, with many underlying mechanisms remaining to be elucidated.

ICIs have achieved notable success in melanoma treatment; however, more than half of patients with advanced melanoma show poor responses to immunotherapy [34]. The overall expression of the Stage 4 SKCM identified co-regulated genes could stratify patient survival outcomes in the TCGA SKCM cohort. Using the LMT classifier model, the expression levels of STAT1, AC139887.1, CCDC183-AS1, and AC022217.3, identified in Stage 4, may further provide a certain degree of distinction between responders and nonresponders to immunotherapy. Additionally, driver genes identified from the coregulated genes across all SKCM stages are differently expressed in different immunotherapy response groups. Liu *et al*. demonstrated that CCDC183-AS1 knockdown significantly suppresses cellular proliferation, colony formation, migration, and invasion capabilities in breast cancer [35]. Lin *et al*. identified SENP1 as playing an oncogenic role across multiple cancer types by promoting tumor cell proliferation, metastasis, and drug resistance [36]. Mao *et al*. revealed that PLAC8 facilitates cancer cell proliferation and modulates immune responses through regulation of PD-L1 ubiquitination [37]. Yang *et al*. reported that combination therapy employing toosendanin (TSN) with ICIs achieved complete therapeutic responses in over 50% of treated murine models [38]. This evidence suggests that these genes may influence patient responses to immunotherapy.

In summary, we characterized the dynamic changes in the synergistic interactions between ICPs and lncRNAs during cancer progression and explored the roles of coregulated genes in survival outcomes and immunotherapy responses in SKCM. Our study paves the way for further investigation into the functions and mechanisms of lncRNAs in immune regulation, particularly in the context of ICP modulation in tumors. Future research is needed to further elucidate the immunological roles of lncRNAs in cancer and their potential applications in immunotherapy.

## Conclusion

This study utilized a PPI network to integrate manually curated ICPs and high-confidence lncRNA-mRNA interaction data, constructing and identifying stage-specific lncRNA-ICP synergistic pairs across multiple cancer types. Several key genes, including MALAT1 and CRNDE, were extensively involved in cancer progression and exhibited distinct regulate patterns across various cancers. The genes identified in the synergistic network were not only associated with the dynamic changes of immune cells during cancer progression but also demonstrated potential for distinguishing patient survival outcomes and responses to immunotherapy. These findings underscore the critical role of lncRNAs in regulating cancer immune responses and provide a foundation for understanding the dynamic synergistic relationships between lncRNAs and ICPs during cancer progression.

**Key Points**

- The synergistic relationship identification framework was based on changes in correlation coefficients between long noncoding RNAs (lncRNAs) and immune checkpints (ICPs) during cancer progression, identifying lncRNA-ICP pairs with significantly high co-expression relationships at specific cancer stages.
- Different cancers at different stages exhibited varying preferences for synergistic modules. Consistency and particularity of regulation patterns for lncRNA-ICP synergistic relationships in cancers were also shown.
- The identified synergistic genes were not only associated with the dynamic changes of immune cells during cancer progression but also demonstrated potential for distinguishing patient survival outcomes and responses to immunotherapy.

## Supplementary Material

supplementary_figures_bbaf370

## Data Availability

All datasets used in this study are publicly available. The analysis results associated with this paper are available on GitHub (https://github.com/LcyWorkSpace/Lnc_icp).
