# Supplementary material for: Dynamic changes of synergy relationship between lncRNA and immune checkpoint in cancer progression
Source: Brief Bioinform. 2025 Jul 29;26(4):bbaf370. doi: 10.1093/bib/bbaf370 (PMC12306437; doi:10.1093/bib/bbaf370)
Supplement: supplementary_figures_bbaf370 [file supplementary_figures_bbaf370.docx]

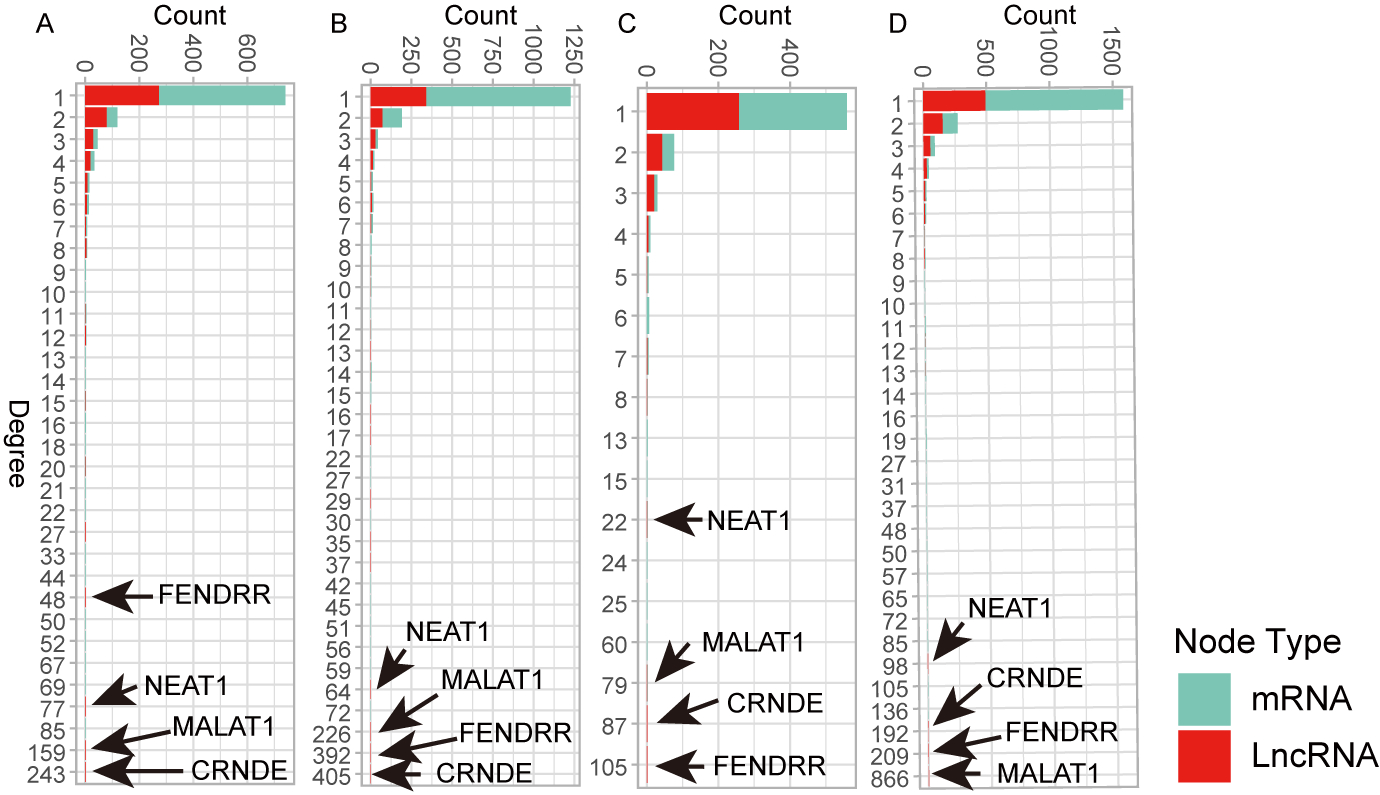


Figure S1. Degree distribution of nodes in the four stage-specific networks.

Alt text: Bar plots show degree distribution of synergistic genes at different stages.


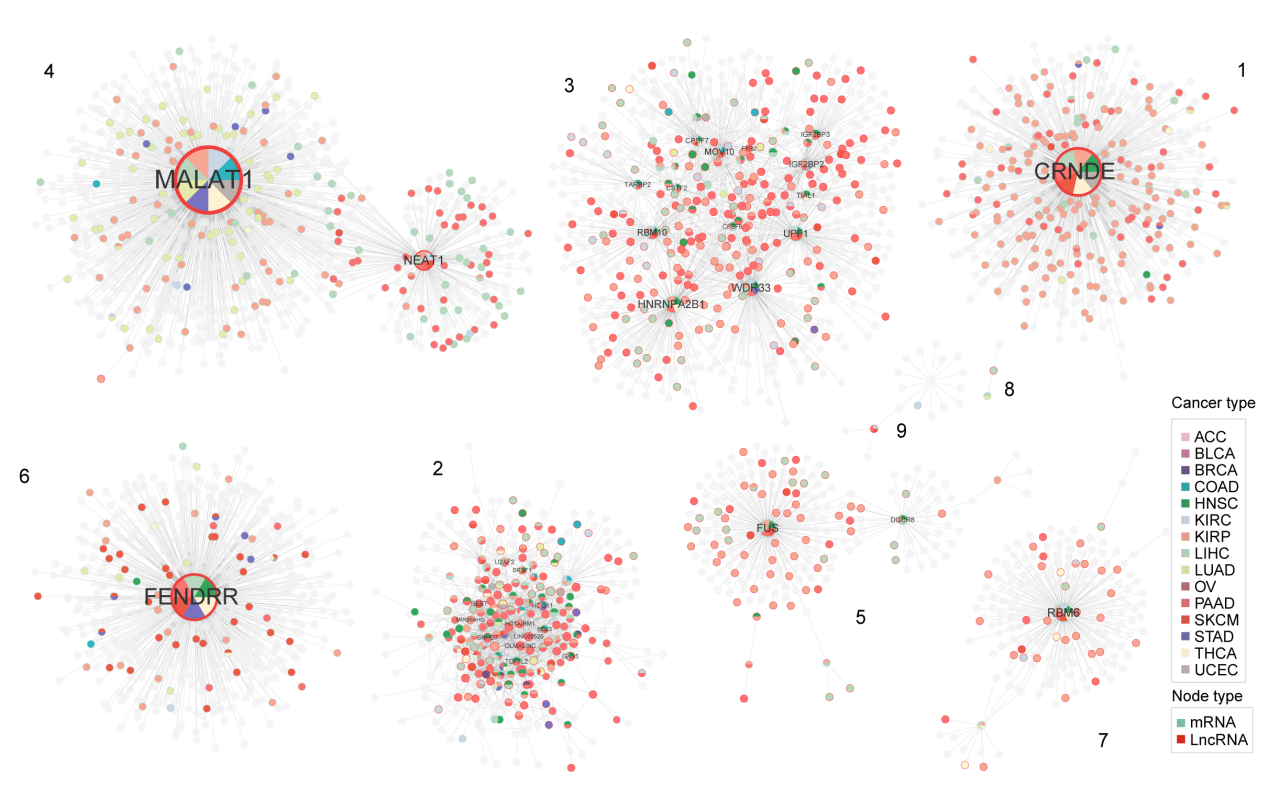


Figure S2. Mapping of the nine modules obtained by GLay clustering in stage one. Nodes labeled with gene names have a degree greater than 10 in this stage.

Alt text: Network of synergistic relationships identified across all cancers in stage 1.


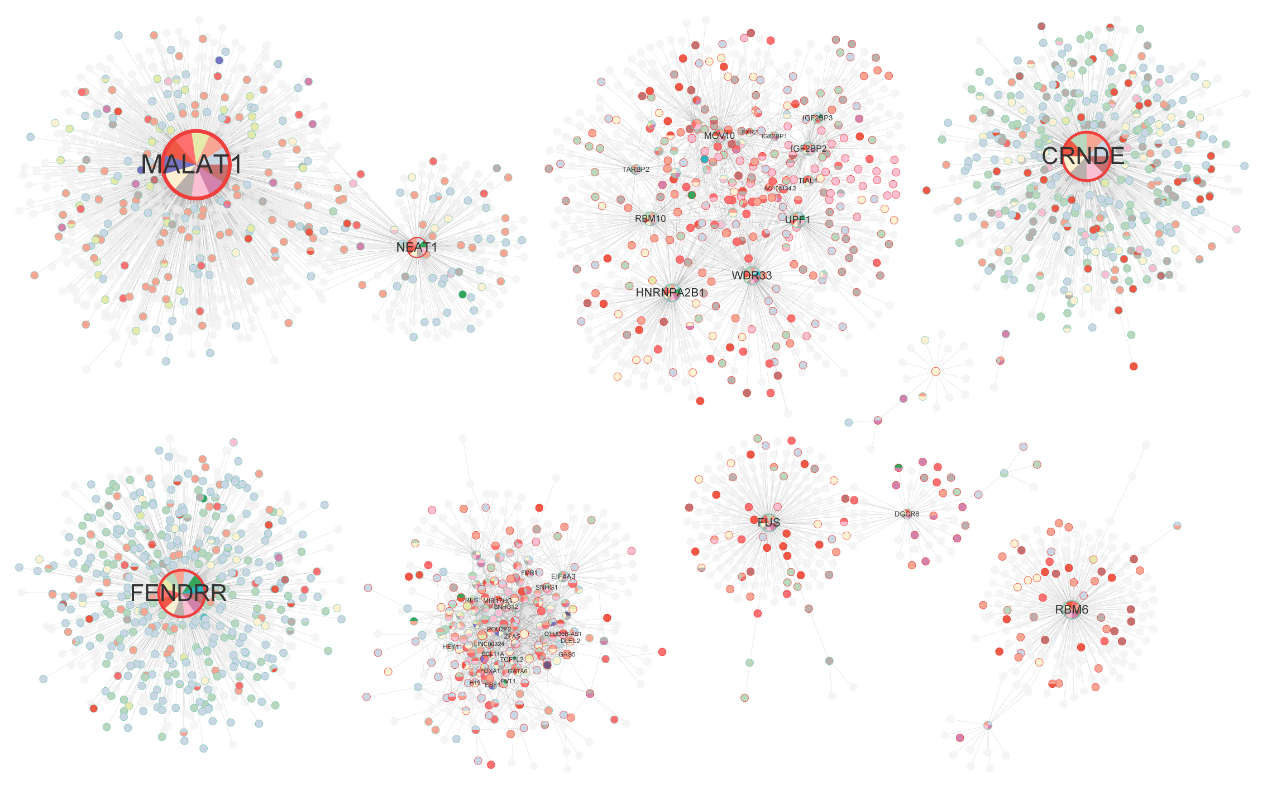


Figure S3. The nine modules obtained by GLay clustering in stage two.

Alt text: Network of synergistic relationships identified across all cancers in stage 2.

**
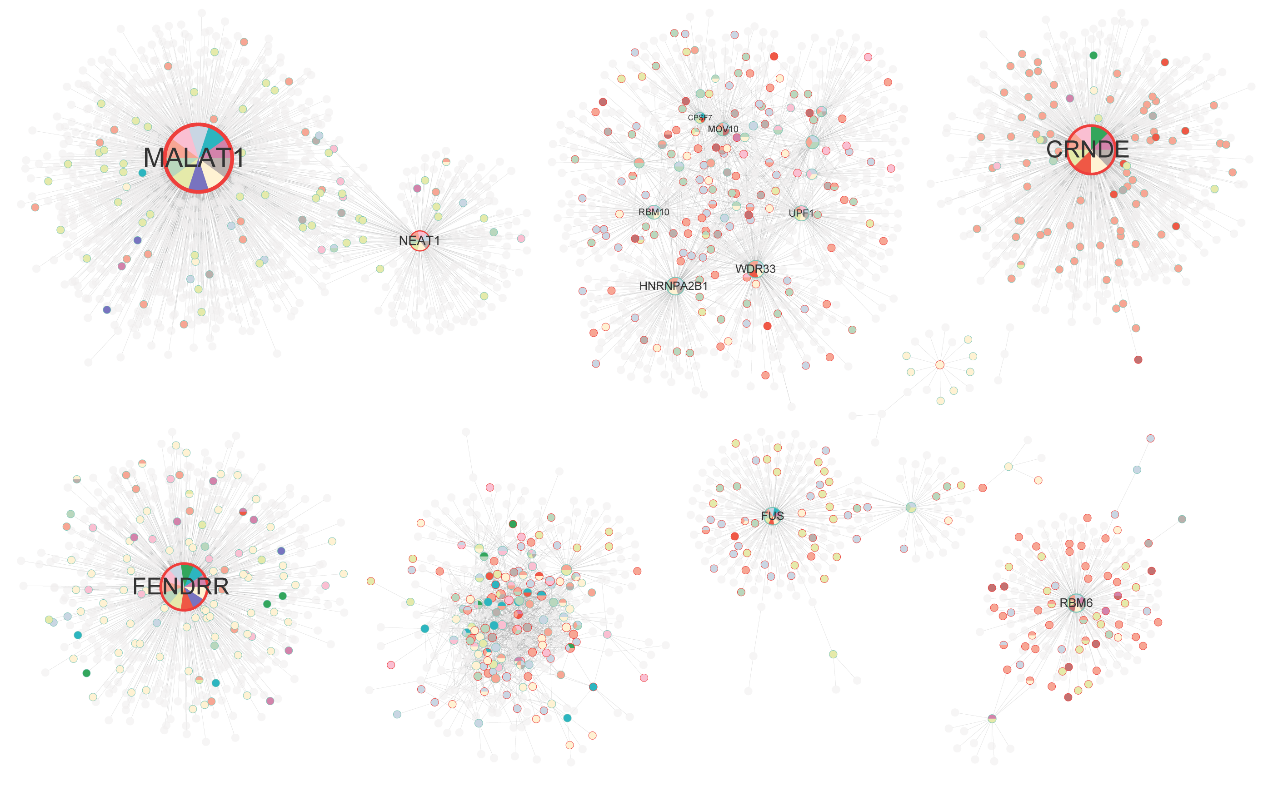
**

Figure S4. The nine modules obtained by GLay clustering in stage three.

Alt text: Network of synergistic relationships identified across all cancers in stage 3.


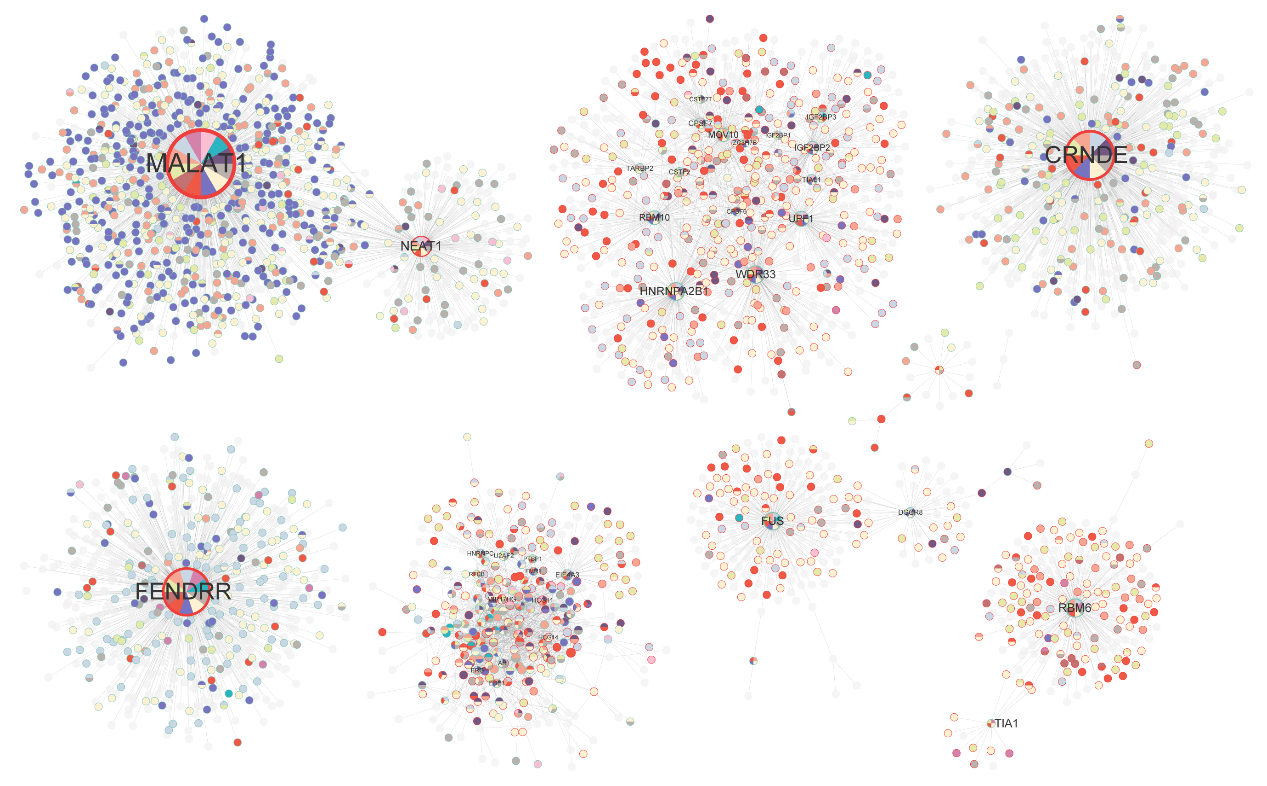


Figure S5. The nine modules obtained by GLay clustering in stage four.

Alt text: Network of synergistic relationships identified across all cancers in stage 4.


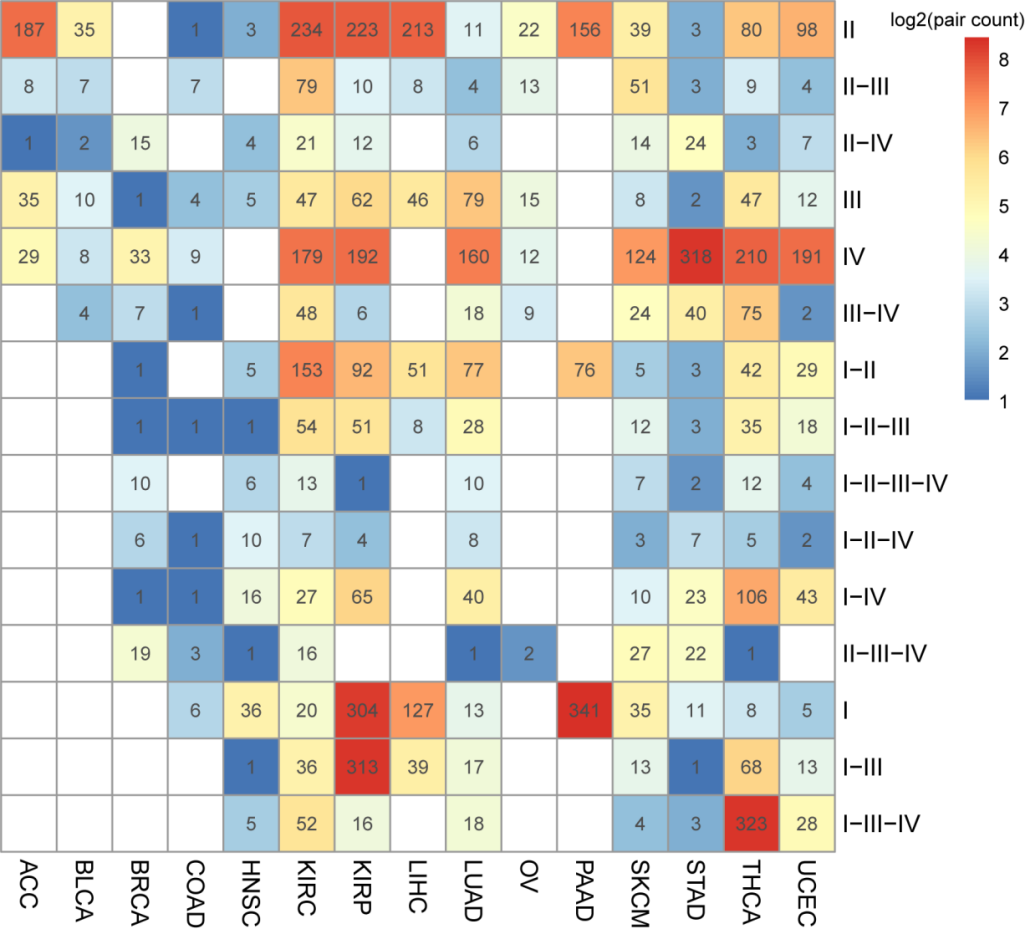


Figure S6. Distribution of co-expression occurrence in multiple stages for synergistic pairs in cancers.

Alt text: Heatmap shows significantly conserved co-expression pairs in multiple stages.


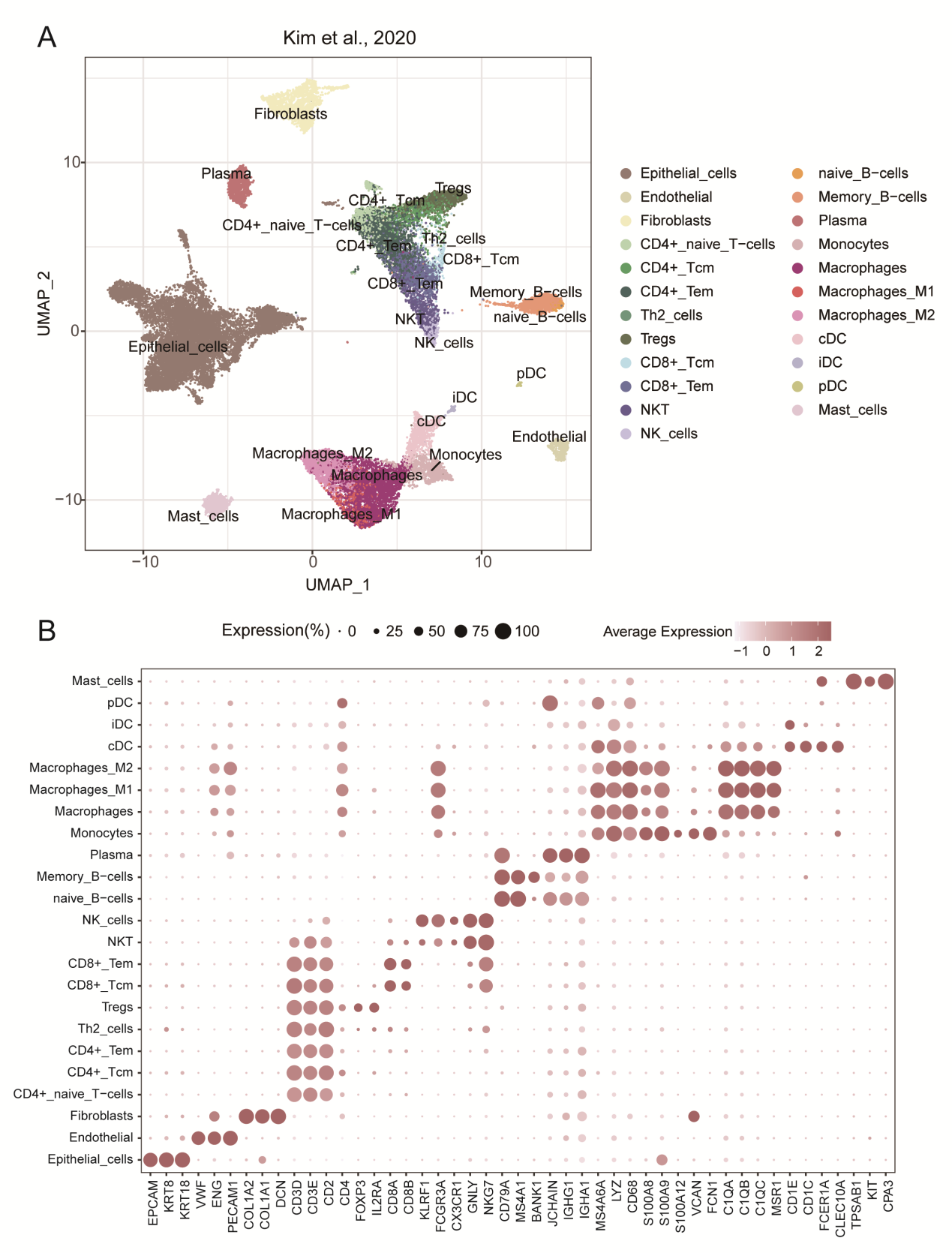


Figure S7. UMAP and cell annotation of single-cell data.

Alt text: Graphs labelled from a to b, including UMAP visualization of cell subclusters and their marker genes.


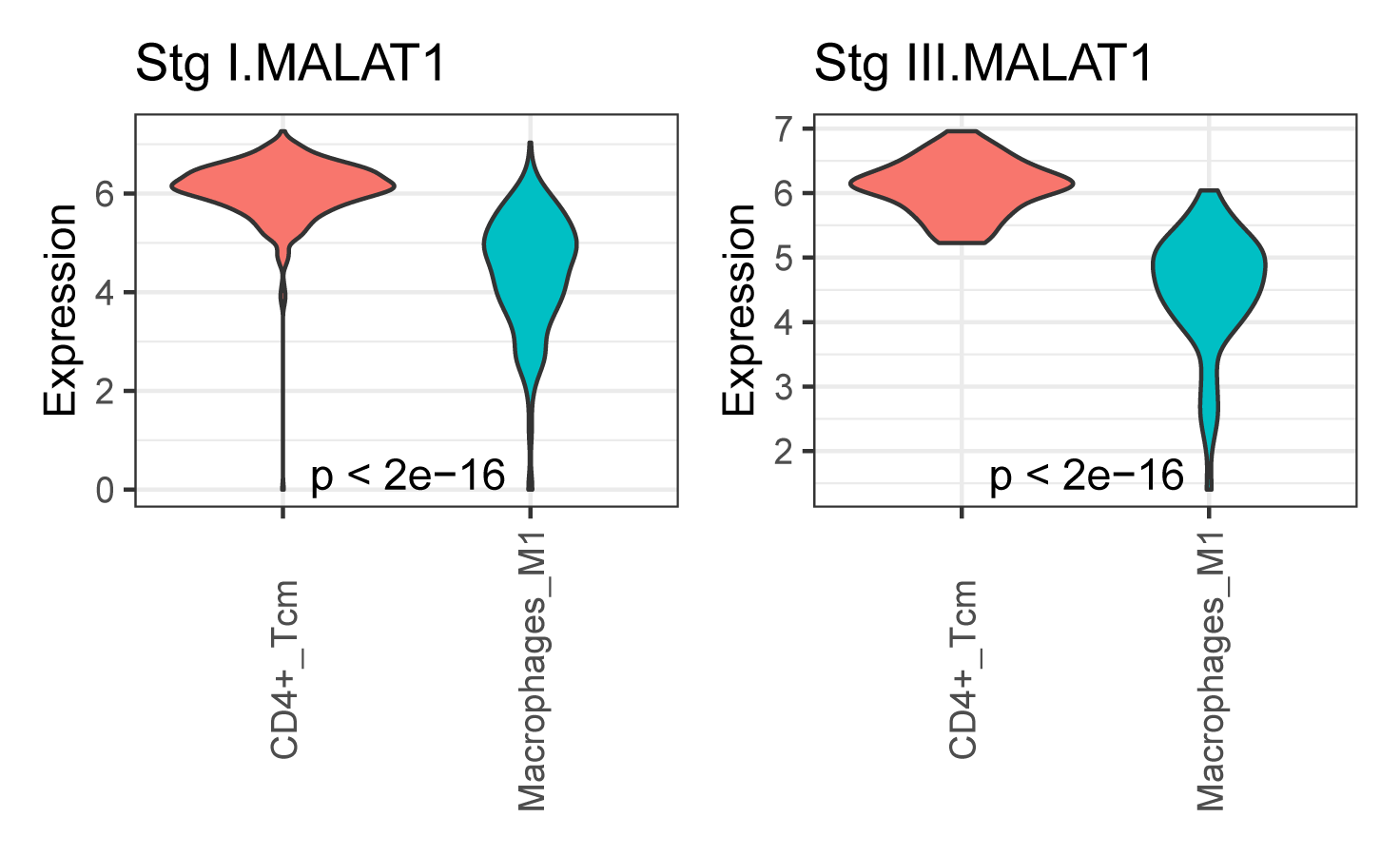


Figure S8. Expression of MALAT1 in CD4+ Tcm and M1 Macrophage.

Alt text: Violin plots show expression of MALAT1 in different stages of two cell types.


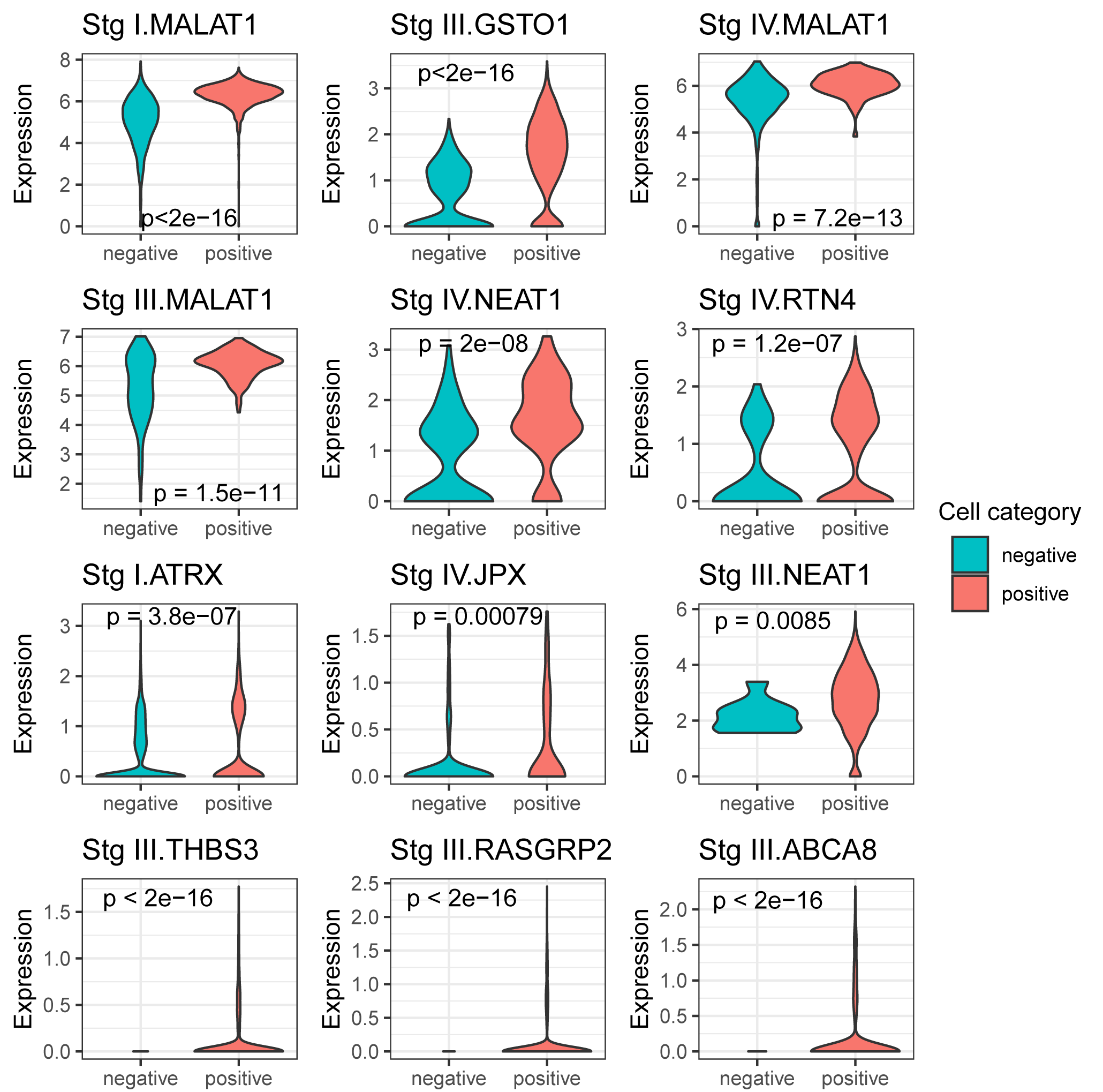


Figure S9. Expression of genes in positive and negative cells.

Alt text: Violin plots show expression of the genes in cell types showing positive/negative correlation in different stages.


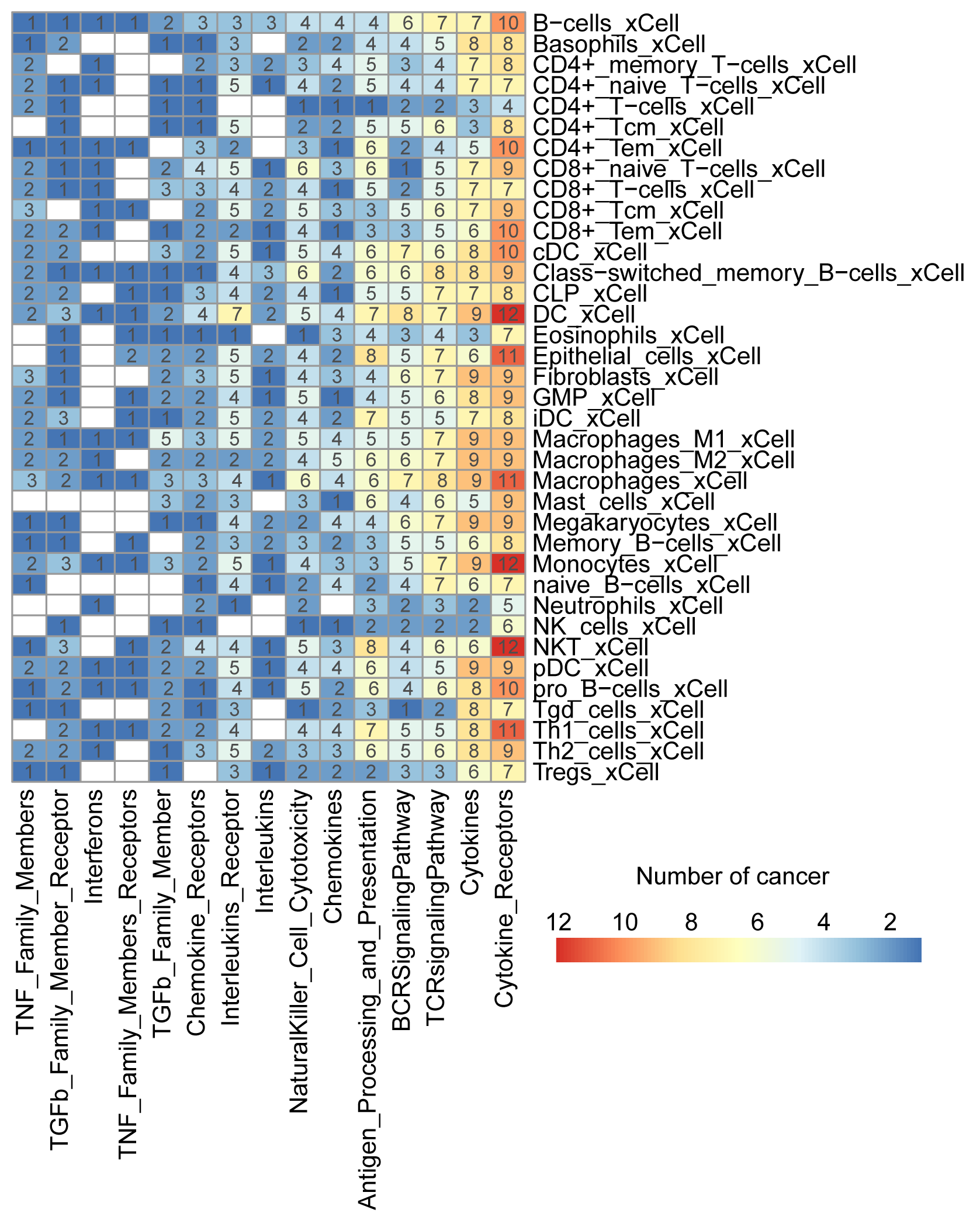


Figure S10. Functional annotation of cell-associated genes in cancers.

Alt text: Heatmap shows functional annotation of genes associated with cell type enrichment scores.


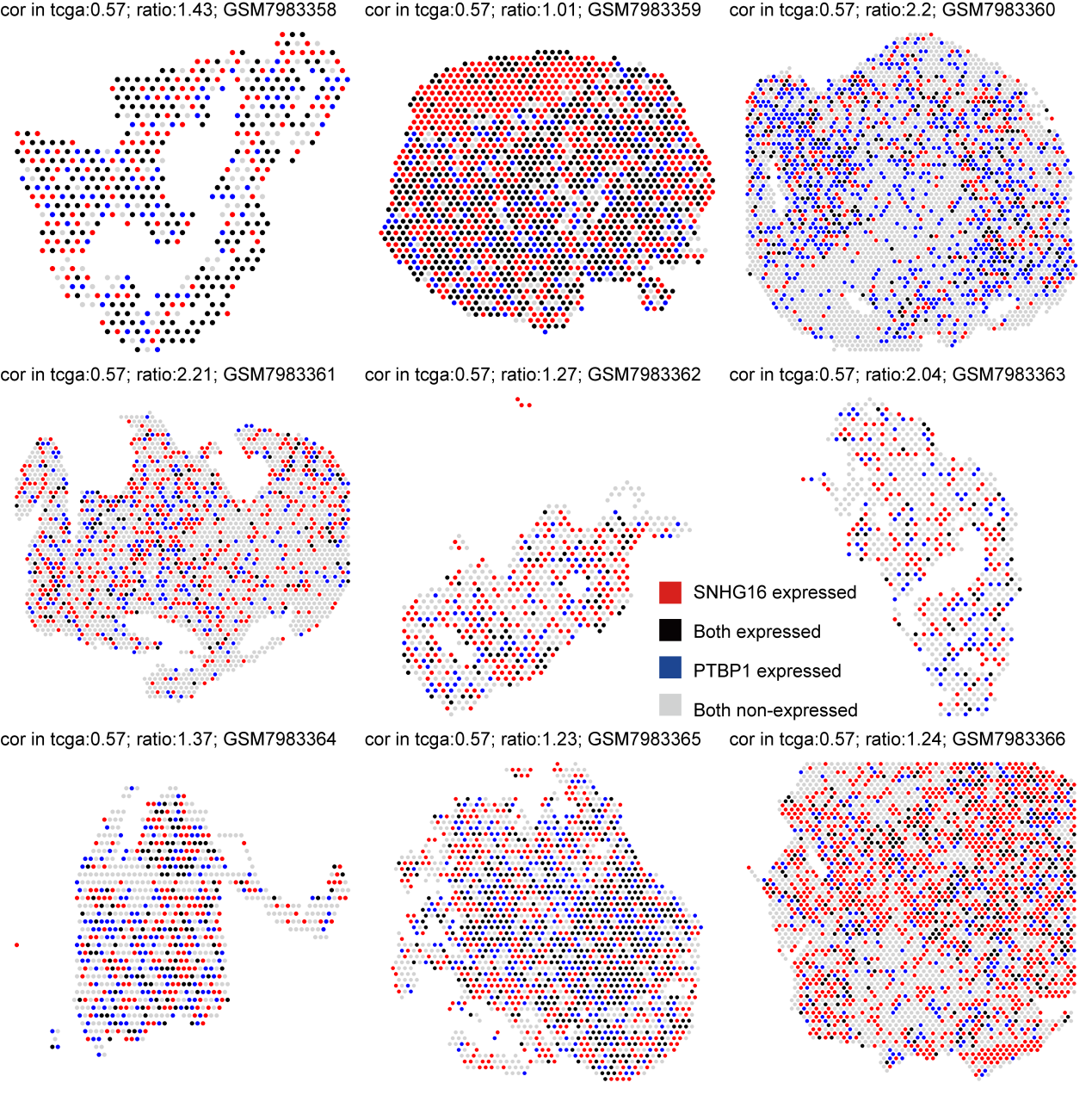


Figure S11. Spatial distribution of SNHG16 and PTBP1.

Alt text: Spatial distribution of synergistic pair across tissue slices.
